# Pine marten predation of common goldeneye nests: Effects of cavity age and habitat override any effect of microtine rodent abundance

**DOI:** 10.1002/ece3.10643

**Published:** 2023-10-24

**Authors:** Geir A. Sonerud, Helge E. Grønlien, Ronny Steen

**Affiliations:** ^1^ Faculty of Environmental Sciences and Natural Resource Management Norwegian University of Life Sciences Ås Norway; ^2^ Anders Bjørnsgårds vei 7 Fåberg Norway

**Keywords:** alternative prey hypothesis, cavity age, forest edge, long‐term spatial memory, microtine rodents, nest box, nest predation

## Abstract

According to the alternative prey hypothesis (APH), the temporal synchrony in population fluctuations of microtine rodents and other small herbivores in boreal areas is caused by generalist predators with numerical and functional response to microtines, leading to an increased predation of prey alternative to microtines in the low phase of the microtine population fluctuations. The tree‐climbing pine marten (*Martes martes*) is a food generalist that includes bird eggs among its alternative prey, also eggs of the cavity‐nesting common goldeneye (*Bucephala clangula*). We used long‐term data to test whether pine marten predation of goldeneye eggs in nest boxes varied as predicted by the APH. As a measure of microtine abundance at the time of nesting, we applied two measures. First, for goldeneye nests located <40 km from our microtine trapping site, we applied the trapping index of microtine rodents. Second, to also use data from nests located >40 km from our microtine trapping site, and from nests in years when trapping was not conducted, we used two proxies for the microtine abundance: whether boreal owls (*Aegolius funereus*) nested in any of our boxes <40 km from each goldeneye nest and the average clutch size of these boreal owls. The probability of predation of a goldeneye nest was independent of the microtine trapping index and independent of the proxies for microtine abundance. However, it increased with cavity age, taken as the number of nesting seasons elapsed since the actual nest box was installed, and declined with distance from habitat with forest canopy. The effect of cavity age confirms that the long‐term spatial memory of pine marten is an important factor in the pattern of its predation on nests in tree cavities.

## Graphical abstract

In a long‐term dataset, the probability of pine marten predation of goldeneye nests was independent of microtine rodent abundance, but not inversely related to it as predicted by the alternative prey hypothesis. However, the probability of nest predation increased with cavity age and declined with distance from habitat with forest canopy. The effect of cavity age confirms that the long‐term spatial memory of pine marten is an important factor in the pattern of its predation on nests in tree cavities.

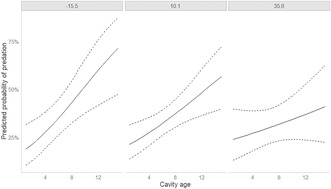

## 1 INTRODUCTION

The alternative prey hypothesis (APH) states that the temporal synchrony between the 3–4 years periodic population fluctuations of microtine rodents and those of other small herbivores, in particular species of grouse, in Fennoscandia is due to generalist predators showing numerical and functional responses to the abundance of microtine rodents, their main prey (Angelstam et al., 1984, 1985; Hagen, 1952). These responses would lead to an increased predation of grouse and several other types of prey alternative to microtines in the low phase of the microtine population fluctuations (Angelstam et al., 1984, 1985; Hagen, 1952). This is equivalent to explanations for predator–prey relationship and prey population dynamics in resource pulse‐driven systems in general, based on functional response in predators and effects on alternative prey (Schmidt & Ostfeld, 2008).

In the APH, the red fox (*Vulpes vulpes*) and the pine marten (*Martes martes*) have been regarded as the most important predators, but the effect of each could not be disentangled in predator removal experiments (Marcström et al., 1988, 1989; see also Lindström et al., 1987; Kurki et al., 1997). However, the decline of the red fox population caused by an irruption of the sarcoptic mange (*Sarcoptes scabei*) in Sweden and Norway in the 1970s revealed that the effect of the red fox on alternative prey was according to the APH (Lindström et al., 1994), and also that the red fox limited the pine marten population (Lindström et al., 1995; Smedshaug et al., 1999). The latter suggested that the pine marten is a less important predator on grouse than the red fox (Smedshaug et al., 1999).

Recently, the effect of the red fox on the population dynamics of willow grouse (*Lagopus lagopus*) was confirmed to be as predicted by the APH (Breisjøberget et al., 2018). There is, however, no study clearly demonstrating such an effect of pine marten on grouse. Jahren et al. (2017) found that pine marten predation on black grouse (*Tetrao tetrix*) nests varied according to the APH when the pine marten density was low and medium, but not when pine marten density was high. Pine marten predation on capercaillie (*Tetrao urogallus*) nests was not as predicted by the APH, but increased with pine marten density. Therefore, Jahren et al. (2017) suggested that the pine marten is a more specialized nest predator than the red fox.

Although consistent with the prediction from the APH, the temporally synchronous population fluctuations in grouse and microtine rodents may also be due to fluctuations in the quality of their common food (Selås, 2006, 2019; Selås et al., 2011). The APH should therefore be tested for alternative prey other than the herbivorous grouse (Selås, 2006). The tree‐climbing pine marten is a predator on bird eggs in general, including those located in tree cavities. Hence, studying the effect of pine marten predation on nests of other birds than grouse would be an opportunity to test the generality of the APH independently of fluctuations in the quality of the food of the small herbivores.

In Finland, Pöysä et al. (2016) found that pine marten predation of nests of a cavity‐nesting duck, the common goldeneye (*Bucephala clangula*), did not vary with the microtine rodent abundance as predicted by the APH and suggested that this may be due to individual martens learning the nest box locations. In Norway, Sonerud (2022) found that pine marten predation of nests of the cavity‐nesting boreal owl (*Aegolius funereus*) did not vary with the microtine rodent abundance as predicted by the APH, but rather increased with cavity age, which suggested that the marten's knowledge of the positions of the cavities overrode any effect of microtine rodent abundance. However, due to the boreal owl's strong numerical response to microtine rodents (Hörnfeldt et al., 2005; Korpimäki & Hakkarainen, 1991; Zarybnicka et al., 2015), it rarely nests in a boreal forest area in years when the microtine abundance there is at its lowest (Sonerud, 1985a) and pine marten predation according to the APH would be highest. Therefore, data on pine marten nest predation on a cavity nester that nests also in years with low microtine rodent abundance, together with data on the age of the nest cavities used, would be needed to confirm the generality of cavity age overriding the effect of microtine rodent abundance in determining pine marten predation of nests in cavities.

Being a diving duck, the common goldeneye, hereafter termed goldeneye, nests independently of the microtine rodent abundance, so data on predation of goldeneye nests would be available also from microtine rodent low years. Therefore, we wanted to use such data to test the APH, and also to control for two important factors in the temporal and spatial variation of pine marten nest predation, namely cavity age and habitat (cf. Sonerud, 2022). In addition, we wanted to test whether boreal owl breeding occurrence and clutch size would be useful as proxies for microtine rodent abundance in tests of the APH for pine marten predation of goldeneye nests.

Pine marten density may affect the probability of predation of goldeneye nests (cf. Jahren et al., 2017). The epizootic of sarcoptic mange among red foxes that spread from the first cases in central Norway in 1975–1976 to the whole country within 10 years resulted in a severe decline of the red fox population (Smedshaug et al., 1999; cf. Lindström et al., 1994). This led to an increase in the hunting bags of the pine marten (Smedshaug et al., 1999), probably due to relaxed competition and predation from the red fox (Lindström, 1989; Lindström et al., 1995; Storch et al., 1990). In our study area, the peak effect of the red fox reduction on the harvest of the pine marten seems to be reached around 1990 (Smedshaug et al., 1999; Sonerud, 2022). Thereafter, the red fox population recovered (Selås, 1998; cf. Breisjøberget et al., 2018), and hunting bags of the pine marten decreased (Sonerud, 2022). However, since the regional hunting bag series do not cover our whole study period, and the national hunting bag series are poorly associated with the regional one from year to year (see Sonerud, 2022 for more details), we refrained from using the pine marten hunting bag as a variable in the analyses.

Here, we extend the study of Pöysä et al. (2016) by demonstrating that pine marten habitat use and spatial memory of tree cavities override any effect of microtine rodent abundance on the probability of pine marten predation of a goldeneye nest. In general, according to the APH, the probability of pine marten predation of a goldeneye nest should be lower when microtine rodents are abundant than when they are scarce, and lower when the microtine abundance has increased since the previous year than when it has decreased. Specifically, we tested the following predictions. First, because pine martens prefer older forest and avoid open habitats such as clear‐cuts (Brainerd & Rolstad, 2002; cf. Sonerud, 1985b), but do not avoid areas with forest cover near clear‐cuts (Angoh et al., 2023), the probability of pine marten predation of a goldeneye nest should decline with distance from the old forest interior into open habitats such as a clear‐cut, either independent of the microtine abundance or in interaction with it. Second, the probability of pine marten predation of boreal owl nests in boxes has been found to increase with time elapsed since the box was installed, a pattern attributed to pine martens memorizing the spatial positions of nest boxes they have found and revisiting the boxes in later breeding seasons (Sonerud, 1985a, 1989, 1993, 2022). It is likely that this pattern would apply to predation of goldeneye nests as well, because goldeneyes nest in the same kind of cavities, including nest boxes, as do boreal owls. If the APH applies to pine marten predation of goldeneye nests, the effects of habitat and cavity age should be affected by microtine abundance, but if the APH does not apply, the effects of habitat and cavity age should hold independent of microtine abundance.

## 2 METHODS

### 2.1 Study area

**FIGURE 1:**
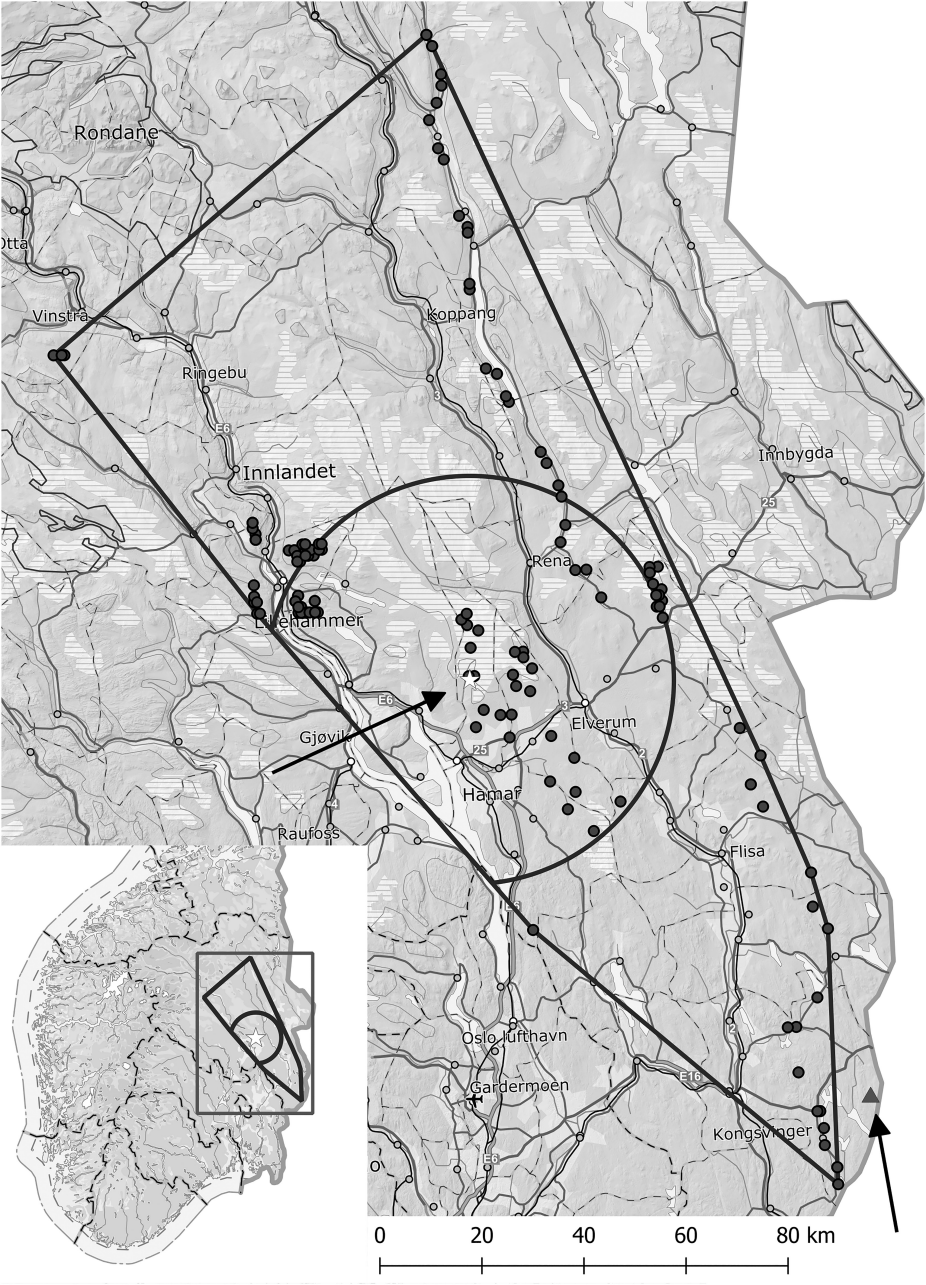
Map of southeast Norway showing the extent of the study area as a minimum convex polygon including the boxes used by goldeneye (filled circles), the site where microtine rodents were trapped (open star), and a circle with radius of 40 km around the microtine rodent trapping site. A microtine rodent trapping site (Wegge & Rolstad, 2018) outside the study area is shown by a filled triangle. The two microtine rodent trapping sites are pointed by arrows. In the cases where two or more nest boxes were closer to each other than 500 m, only one is shown. The small open circles denote towns and villages and are not related to the study.

The study was conducted during 1972–2020 between 60°01′‐62°04′ N and 9°38′‐12°23′  E in Hedmark and Oppland counties (from 2020 pooled to Innlandet County) in southeast Norway (Figure 1). The study area is covered by coniferous forest managed by modern forestry techniques, that is, harvesting by clear‐cutting, regeneration by planting, and thinning by selective cutting. It overlaps to a large extent with the study area of Sonerud (2022). The convex polygon circumscribing the nest boxes spans an area of c. 14,000 km^2^ (Figure 1). The elevation of the nest boxes used by goldeneye ranged 170–890 m a.s.l., with median = 400 m and average = 447 ± 13 m (*n* = 181).

### 2.2 Study species

The goldeneye is a medium‐sized diving duck (female body mass c. 700 g) that occurs over large parts of the Holarctic boreal forest (Cramp & Simmons, 1977). In the western Palearctic, goldeneyes nest mostly in cavities excavated by the black woodpecker (*Dryocopus martius*) (e.g., Cramp & Simmons, 1977), but readily accept nest boxes (e.g., Dow & Fredga, 1983). Goldeneyes are exposed to a significant risk of nest predation by the pine marten (Dow & Fredga, 1983, 1985; Pöysä, Milonoff, & Virtanen, 1997), which is a medium‐sized (c. 1 kg) mustelid with a relatively large home range (on average 7 km^2^ at 60° N in Sweden and Norway) and a generalist diet (Brainerd, 1997; Helldin, 1999). The pine marten visits tree cavities year‐round and uses them for roosting, denning, and food storing (Brainerd et al., 1995; Sonerud, 1985b), and takes any prey that may happen to be there, including eggs and nestlings. The positions of cavities are probably learned (Elmberg & Pöysä, 2011; Pöysä, 2006; Sonerud, 1985a, 1989, 1993, 2022), and the pine marten spends the most time on the ground and preys mainly on small mammals, in Fennoscandia microtine rodents (Helldin, 2000; Pulliainen & Ollinmäki, 1996).

Goldeneyes migrate to the nearest coast or longer, and are absent from most of the breeding grounds during winter, except when or where lakes or rivers remain ice‐free throughout the year (Cramp & Simmons, 1977). In our study area, they are absent from around September to April. The male is not involved in nest site selection or parental care, and the female has no nesting territory, so the brood‐rearing site may be far away from the nest site (Dow & Fredga, 1985; Paasivaara & Pöysä, 2008; Pöysä & Virtanen, 1994; Pöysä, Virtanen, & Milonoff, 1997). The ducklings leave the nest within 2 days after hatching, and the female does not return to the nest thereafter (Cramp & Simmons, 1977; Pöysä, 2006). After having been successfully used for nesting by goldeneye, the nest cavity contains eggshell membranes and fragments of the hatched eggs as well as the down that the female has plucked and used for covering the eggs during incubation and recesses (Eadie & Gauthier, 1985; Pöysä et al., 2014).

### 2.3 Nest boxes and nests

New boxes were installed and added to the existing population of nest boxes most years. Most boxes were installed c. 5 m above ground. In comparison, cavities excavated by the black woodpecker in Sweden were on average 7 m above ground, (Johnsson et al., 1993; Rolstad et al., 2000). When installed, all boxes were lined with a 5–10 cm deep layer of fine wood shavings covering the bottom. Boxes were not systematically cleaned after each nesting. Cleaning of a nest box does not affect the probability of reuse by goldeneye (Sonerud, 2021a).

Most boxes were installed in single trees in clear‐cut areas or other open habitats, or in trees in edges between old forest and clear‐cuts or other open habitats, and fewer in habitats with forest canopy. This reflected the habitat preferences of black woodpeckers selecting a tree in which to excavate a nest cavity (see Rolstad et al., 2000) and made the boxes attractive for goldeneyes as well (cf. Pöysä et al., 1999). Before 2005, the distance to lakes and rivers was random, but later, most boxes were installed within 100 m of lakes. Of the 181 boxes used for nesting by goldeneyes in this study, 109 were situated in a clear‐cut or another open habitat, 50 in the edge between old forest and clear‐cuts or other open habitats, and 22 within an old forest stand.

Each box was usually visited several times between March and July each year to record the outcome of nesting attempts by goldeneye, that is, whether the nest was predated. We defined a box as being selected when at least one goldeneye egg had been laid there. A goldeneye nesting attempt was classified as successful when eggshell membranes and fragments of the hatched eggs as well as the down were found.

We classified a nesting as predated when either down was found, without any trace of eggs (149 cases), or when broken eggs or eggshells without membranes were found (24 cases). In 8 of these 173 cases, we found that the goldeneye female had been killed. In 34 cases of nest predation, we found evidence of pine marten being the predator, such as hairs in the nest box entrance, scats on the roof of the nest box, claw mark on the outside walls of the nest box, or a trail of goldeneye down on the tree trunk, left as the marten climbed down after having been inside the box. In the remaining majority of cases where we were unable to identify the predator, we had no reason to believe that any other predator than the pine marten was involved.

The problem of underestimating nest predation by failure to include nests already predated was minimized by our knowledge of potential nest sites. However, if a goldeneye nest had been predated prior to the first nest box check for the season, and before the goldeneye female had started to pluck the down used for covering the eggs during incubation and recesses, which on average starts when 3 eggs have been laid (H. E. Grønlien and G. A. Sonerud, unpublished data), and all eggs had been removed, the nesting attempt may have been overlooked rather than classified as predated. Because a number of goldeneye nests were abandoned without being predated, we avoid using the term successful nest as a contrast to predated nest, and rather use the term non‐predated nest or nest that avoided predation.

We were able to classify whether the nest was predated or not for a total of 509 nesting attempts by goldeneye. The altitude of these nests ranged 170–890 m, with median = 421 m and mean ± SE = 466 ± 8 m. Of these nests, 289 were situated in open habitats (mainly clear‐cuts), 58 in habitats with a forest canopy (spruce or pine forest), and 162 at the edge between open habitat and forest canopy habitat.

Of the 181 nest boxes, 65 were used only once, 47 twice, and 69 were used 3–11 times (Table S1). Of the 509 nesting attempts, 156 were within 40 km from the microtine rodent trapping site in the years when trapping was conducted (see Section 2.4). These 156 nesting attempts were in 62 nest boxes, of which 24 were used only once, 19 twice, and 18 were used 3–8 times (Table S1).

The 509 nesting attempts were distributed over 47 years, of which 5 years had only one nesting attempt, 6 years had two nesting attempts, and 36 years had 3–31 nesting attempts (Table S2). The 156 nesting attempts within 40 km from the microtine trapping site in the years when trapping was conducted (see Section 2.4) were distributed over 34 years, of which 2 years had only one nesting attempt, 8 years had two nesting attempts, and 24 years had 3–11 nesting attempts (Table S2).

### 2.4 Microtine rodent abundance

**FIGURE 2:**
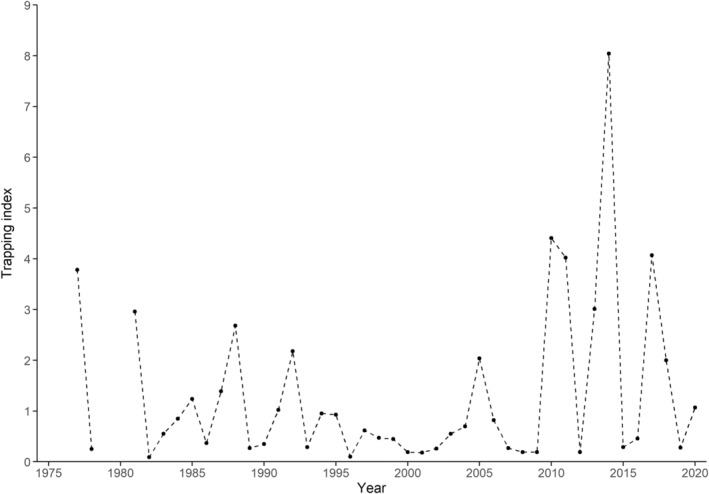
Microtine rodent spring trapping index, taken as number of animals (bank vole (*Clethrionomys glareolus*), field vole (*Microtus agrestis*), tundra vole (*Microtus oeconomus*), and wood lemming (*Myopus schisticolor*) combined) trapped per 100 trap nights during 1977–1978 and 1981–2020 in the central part of the study area (see Figure 1).

In 1977–1978 and 1981–2020, microtine rodents were trapped at the same site each spring as soon as the snow cover had disappeared, which varied from early May to early June. The trapping site was situated 550–600 m a.s.l. in the boreal forest at 60°56′ N, 11°08′ E in southeast Norway (Figure 1, see also Sonerud (1986)). In each trapping session, c. 300 wooden snap traps (brand Rapp, Nordenfjeldske Børstefabrikk, Norway) baited with cocoa fat (brand Delfia, Mills AS, Norway) were put out and checked each morning for 4 days (for more details, see Sonerud (2022)). The number of trap nights ranged 1000–1184, with median = 1095 and mean ± SE = 1096 ± 6 (*n* = 42). A microtine rodent trapping index was calculated as number of animals of all species combined (bank vole (*Myodes glareolus*), field vole (*Microtus agrestis*), tundra vole (*Microtus oeconomus*), and wood lemming (*Myopus schisticolor*)) trapped per 100 trap nights. This index ranged a 90‐fold (0.09–8.04, Figure 2). As an index of the year‐to‐year change in microtine abundance, the trapping index of the previous year was subtracted from the trapping index of the current year. Although an expected peak in 2001 did not appear (Figure 2), the microtine rodent population fluctuations were overall as required for testing the APH.

### 2.5 Boreal owl nest occurrence and clutch size as proxies of microtine rodent abundance

Population fluctuations of microtine rodents tend to be synchronized over large areas, but the extent of this synchronization is poorly documented. A study performed over almost 300 km along the east side of our study area (see Figure 1) during 1990–1994 found that local populations of the bank vole <30–40 km apart exhibited statistically significant synchrony in growth patterns (Steen et al., 1996). It later turned out that this estimate was obtained in a period with generally low amplitudes and low spatial synchrony of bank vole populations in Hedmark County (Selås et al., 2021). Prior to 1990 and after 2003, bank vole populations at two sites in Hedmark County located 120 km apart (the trapping site in our study and the trapping site of Wegge and Rolstad (2018); see Figure 1) fluctuated in synchrony (Selås et al., 2021). Still, for the whole study period, we used 40 km as a conservative limit for the range within which the microtine abundance at the trapping site was representative (see Figure 1). Thus, for goldeneye nests located >40 km from the microtine trapping site, we were unable to use the microtine trapping index as a measure for the microtine abundance.

The goldeneye nests sympatrically with the boreal owl (Cramp, 1985; Cramp & Simmons, 1977), and these birds may even use the same cavity in alternate years (G. A Sonerud and H. E. Grønlien, unpublished data). However, whereas goldeneyes are found nesting each year in any part of our study area, boreal owls are able to nest only in 1–2 years of the 3–4 years vole cycle in a specific part of the study area (e.g., Sonerud, 1985a). Because the boreal owl shows a strong numerical response to microtine rodents in Fennoscandia (Hörnfeldt et al., 2005; Korpimäki & Hakkarainen, 1991; Sonerud, 2022; Zarybnicka et al., 2015), its absence or presence as nesting in an area can be taken as a simple proxy of low or high, respectively, microtine rodent abundance in the area during the breeding season. A refined proxy of the microtine abundance in the area in the breeding season would be the clutch size of the boreal owl. We refrained from using the number of nest boxes occupied by boreal owl within an area as a refined proxy of the microtine abundance, because the probability of use of a nest box by boreal owl declines with time elapsed since the box was installed (Sonerud, 1985a, 2021b), and boxes available for boreal owls in our study area had cavity age (see Section 2.7) ranging from 1 to >10 (Sonerud, 2022). Thus, the number of nest boxes occupied by boreal owl within an area would be affected by the age distribution of the nest boxes in that area.

For each nesting attempt of the goldeneye, we assigned an indirect measure of the abundance of microtine rodents in two ways. First, we decided whether there was any boreal owl nesting in our nest boxes in the same year within a distance of 40 km, that is, within an area of c. 5000 km^2^ around the actual goldeneye nest (see Sonerud (2022) for information on nest box checking and definition of a boreal owl nesting). This presence/absence (1/0) could be calculated for all goldeneye nests (*n* = 509). For each goldeneye nest with data on boreal owl presence/absence = 1 (*n* = 397), the number of boreal owl nests ranged 1–26 (median = 4, average = 4.7 ± 0.2).

Second, for each nesting attempt of the goldeneye, we calculated the average clutch size of boreal owls nesting in our boxes within 40 km of the actual goldeneye nest in the same year (range: 2.0–7.0, median = 4.8, mean = 4.9 ± 0.05, *n* = 333). The number of boreal owl nests from which this average was calculated ranged 1–24 (median 3, average 4.2 ± 0.2) for each goldeneye nest. Because nest predation was substantial (cf. Sonerud, 1985a, 2022), boreal owl nests were often found predated at the first visit, leaving the clutch size unknown. If this was the case for all known boreal owl nests within 40 km from a goldeneye nest in the actual year, the average boreal owl clutch size for this goldeneye nest was scored as unknown (*n* = 64). If no boreal owls were found nesting within 40 km from a goldeneye nest, the boreal owl clutch size for this goldeneye nest was scored as zero (*n* = 112). Thus, the variable expressing boreal owl clutch size was scored for fewer goldeneye nests (*n* = 445) than the variable expressing whether or not boreal owls were nesting (*n* = 509).

The effect of microtine rodent abundance on pine marten predation of goldeneye nests may not only be due to the current abundance of microtine rodents, but also to the change in abundance since the previous year. Therefore, we calculated two proxies for this change. First, for each nesting of the goldeneye, we used the measure of whether any boreal owl nested in our nest boxes within a distance of 40 km in the current year and subtracted the corresponding measure for the previous year. In this way, we obtained a proxy (*n* = 508) for whether the microtine rodent abundance had declined since the previous nesting season (*n* = 98), increased since the previous nesting season (*n* = 96), or remained low or high (*n* = 314). Second, for each nesting of the goldeneye, we subtracted the average clutch size of boreal owls found nesting within 40 km in the previous year from the average clutch size of boreal owls found nesting within 40 km in the current year (range: −6.5 to 7.0, median = 0.0, mean = 0.13 ± 0.16, *n* = 383), giving a refined proxy for the change in microtine abundance from the previous nesting season to the current one.

### 2.6 Habitat

As a linear measure of habitat relevant for the risk of nest predation, we used the shortest distance from a nest box to the nearest edge between forest and open habitat, the latter being either clear‐cut areas or bog. Nest boxes situated at the edge between forest and open habitat were assigned a value of zero, while boxes situated within a forest stand were assigned a negative value and boxes situated in a clear‐cut or on a bog were assigned a positive value.

Among the 509 goldeneye nesting attempts, the distance to edge ranged from −90 m to 200 m, with median = 5 m. Distance to edge was >100 m in seven cases, and these seven values were set to 100 m. Average distance to edge was then 10 ± 1 m. The distance to edge was marginally non‐significantly longer for nesting attempts <40 km from the microtine trapping site in the years when trapping occurred than for the other nesting attempts (*n* = 156 vs. *n* = 353, 10% percentile 0 vs. −5 m, 90% percentile 43 vs. 38 m, median = 5 m in both cases, Wilcoxon test, *χ* ^2^ = 3.76, df = 1, *p* = .052).

A recent study based on camera trapping in Norway found that pine marten occurrence was higher when old forest was present within 100 m of the camera trap, but did not find a difference between pine marten occurrence when a clear‐cut was present or absent within 100 m (Angoh et al., 2023). We analyzed habitat on the same scale, that is, a box was within 100 m from the edge between forest and clear‐cut on either side.

### 2.7 Cavity age

We scored cavity age for a nest box when used as the number of nesting seasons elapsed since the box was installed in the actual tree, assigning the value 1 for the first nesting season the box was available, 2 for the second season, and so on. Among the 509 goldeneye nesting attempts, the cavity age ranged from 1 to 21, with median = 6. Cavity age was >15 in nine cases, so these nine values were set to 15. The average cavity age was then 6.6 ± 0.2. Cavity age was not significantly different for nesting attempts <40 km from the microtine trapping site in the years when trapping occurred than for the other nesting attempts (*n* = 156 vs. *n* = 353, 10% percentile = 2 in both cases, 90% percentile = 12 in both cases, median = 6.5 vs. 6, Wilcoxon test, *χ* ^2^ = 1.00, df = 1, *p* = .32).

### 2.8 Data analysis

Data preparation and explorative analyses were conducted in JMP® Pro version 15.0.0 (SAS, 2019), while the final analyses were performed using general linear mixed models (GLMM) in R version 4.0.3 (R Core Team, 2020). The unit in statistical tests was each nesting attempt by goldeneye. We used a binomial GLMM (logit link), where the response variable was whether a nest was classified as predated or not. Nest box ID was included as a random effect to account for repeated measurements. The model also accounted for temporal autocorrelation (AR1 structure; package “glmmTMB” (Brooks et al., 2017a, 2017b; Magnusson et al., 2017)) in case a predation event was dependent on the previous nesting outcome in the same nest box.

In models with goldeneye nests situated <40 km from the microtine rodent trapping site in a year when microtine rodents were trapped (42 years), fixed explanatory variables were microtine rodent trapping index (or alternatively year‐to‐year change in microtine index), cavity age, and distance from the nest box to the nearest edge between forest and open habitats. In addition, microtine rodent trapping index was substituted with the fixed variables boreal owl breeding occurrence (whether boreal owls were recorded breeding <40 km from each goldeneye nest) and boreal owl clutch size (average clutch size for boreal owl nests <40 km from each goldeneye nest), and alternatively with year‐to‐year change in boreal owl breeding occurrence and year‐to‐year change in average boreal owl clutch size. The variable for change in boreal owl breeding occurrence was categorized as “negative,” “neutral,” or “positive,” representing different levels of change. “Negative” indicated a decline in owl breeding occurrence, “neutral” indicated no change, and “positive” indicated an increase in owl breeding occurrence. We treated this variable as an ordered factor in the analysis.

In models with nests from the whole study area and from all study years (49 years), for each goldeneye nests, the microtine rodent trapping index was substituted with two proxies: first, boreal owl breeding index for the area <40 km from the actual goldeneye nest, and second, boreal owl average clutch size within this area.

Because the effect of cavity age on the probability of nest predation may depend on the distance to forest edge, we included the interaction between cavity age and distance to edge in all models. In addition, because the effect of cavity age and distance to forest edge on the probability of nest predation may depend on microtine rodent abundance, we included the interaction between microtine abundance (and its proxies) and cavity age and distance to edge in all models.

We compared models of all combinations of the fixed effects using the “dredge” function in the “MuMIn” package (Barton, 2022). Candidate models were ranked using the Akaike information criterion corrected for small sample size (AIC_c_), following recommendations by Burnham et al. (2011) and Richards et al. (2011). We only present models with ΔAIC_c_ <5.0 from the model with the lowest AIC_c_ value. We considered models with ΔAIC_c_ <2.0 to be well supported and thus competing with the model with the lowest AIC_c_ value. Among competing models, the one with the lowest number of effects was considered the most parsimonious. We also report AIC_c_ weight for most models, and use evidence ratio (ER) when comparing some of the models, that is, the ratio between the corresponding AIC_c_ weights (see Burnham et al. (2011), Richards et al. (2011) and Cade (2015) for definitions). We followed the advice by Cade (2015) and refrained from model averaging as well as the use of relative weight of a variable (i.e., the sum of AIC_c_ weights for all models in a model set in which the variable appeared) for evaluating the relative importance of explanatory variables.

For the most parsimonious models and for the highest‐ranked models, we provide parameter estimates based on standardized continuous variables. All estimates are given with ±1 SE.

For graphical presentation, we reanalyzed the actual models using raw data, as untransformed data yield more interpretability than transformed ones.

## 3 RESULTS

The overall probability of predation of a goldeneye nest was 0.34 (*n* = 509).

### 3.1 Effect of microtine rodent spring abundance

**FIGURE 3:**
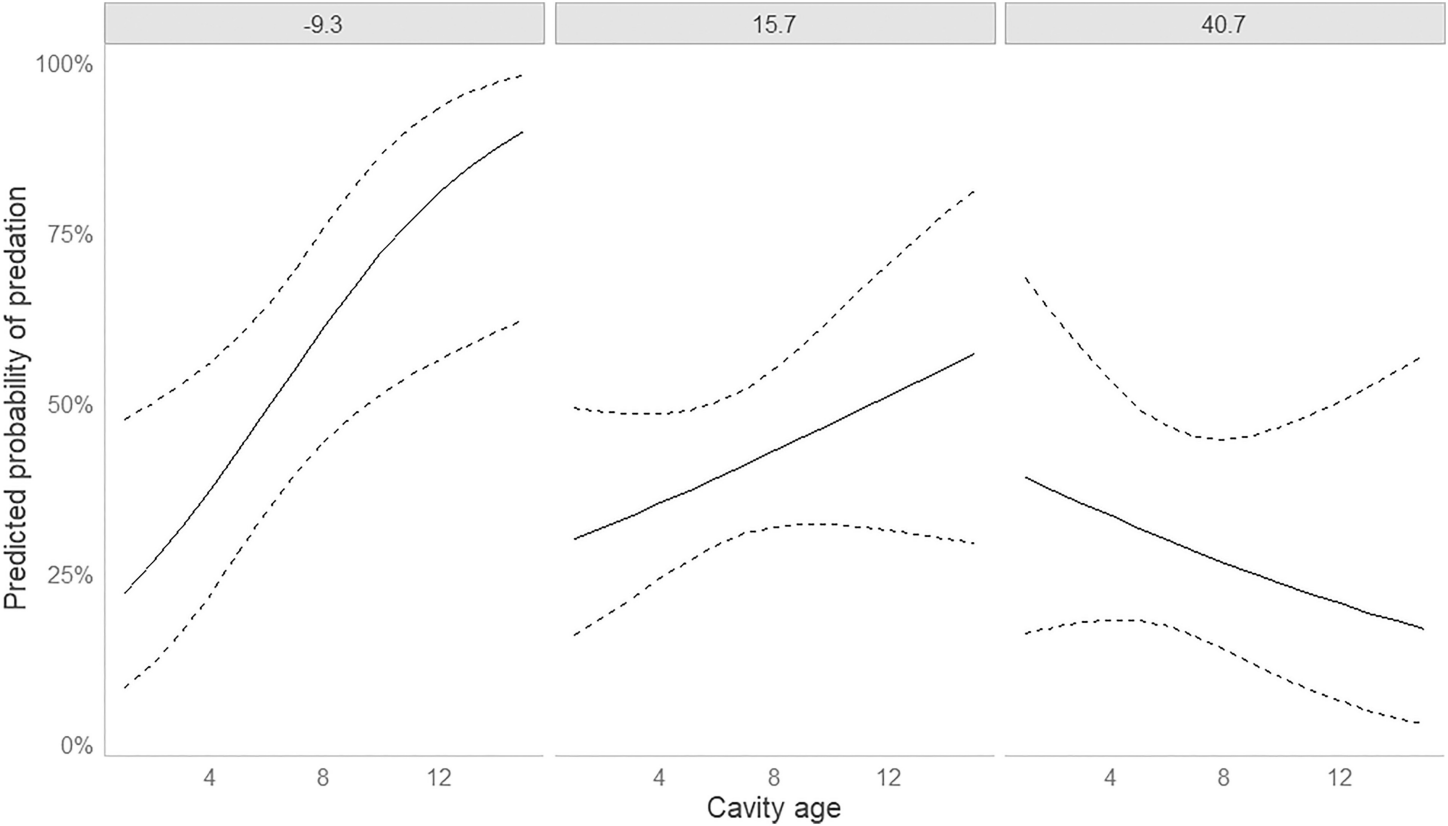
Predicted probability of predation of a goldeneye nest as function of cavity age for three values of distance from forest edge for nests located <40 km from the microtine trapping site in the years when trapping was conducted (*n* = 156). The three values of distance from forest edge are the mean ± 1 SD (−9.3, 15.7, and 40.7 m).

Among the models including microtine rodent trapping index, cavity age, and distance to forest edge, the most parsimonious included only the interaction between cavity age and distance to forest edge (Table S3). The effect of this interaction was significant (Table 1a). The negative interaction between cavity age and distance to forest edge means that the probability of predation as function of cavity age increased more slowly with increasing distance from the forest interior (Figure 3).

**TABLE 1:** Parameter estimates for a subset of GLMM models for the probability of predation of a goldeneye nest situated <40 km from the microtine rodent trapping site in a year when microtine rodents were trapped in relation to the microtine rodent spring trapping index, corrected for the random effect of nest box ID, and temporal autocorrelation of year (*n* = 156 nests, 62 boxes, 34 years).

Explanatory variable	Estimate ± SE	*z*	*p*
*a*			
Intercept	−0.382 ± 0.226	−1.688	.091
Cavity age	0.282 ± 0.224	1.259	.21
Distance edge	−0.537 ± 0.260	−2.070	.038
Cavity age * Distance edge	−0.571 ± 0.250	−2.279	.023
*b*			
Intercept	−0.392 ± 0.221	−1.775	.076
Microtines spring	0.337 ± 0.211	1.596	.11
Cavity age	0.208 ± 0.224	0.929	.35
Distance edge	−0.622 ± 0.268	−2.318	.020
Cavity age * Distance edge	−0.569 ± 0.249	−2.288	.022
*c*			
Intercept	−0.457 ± 0.241	−1.894	.058
Microtines spring	0.278 ± 0.229	1.217	.22
Cavity age	0.291 ± 0.252	1.154	.25
Distance edge	−0.632 ± 0.280	−2.255	.024
Cavity age * Distance edge	−0.585 ± 0.273	−2.143	.032
Microtines spring * Cavity age	0.281 ± 0.249	1.128	.26
Microtines spring * Distance edge	0.130 ± 0.286	0.456	.65
Microtines spring * Cavity age * Distance edge	−0.303 ± 0.308	−0.983	.33

*Note*: (a) The most parsimonious model (ΔAIC = 0.38). (b) The highest‐ranked model (ΔAIC = 0.00). (c) The full model (ΔAIC = 4.95). All models included in this analysis are described in Table S3. Generalized linear mixed‐effect models with log link function, binomial distribution, and Laplace approximation to the likelihood. Continuous explanatory variables are standardized.

A model also including microtine rodent trapping index had higher AIC_c_ weight (Table S3; ER = 1.21 to the most parsimonious model). In this model, the effect of microtine rodent trapping index was not significant, while the effect of the interaction between cavity age and distance to forest edge was significant (Table 1b). The non‐significant effect of microtine index was positive, thus opposite to what was predicted.

In the full model, which had a low AIC_c_ weight (ER = 0.10 to the most parsimonious model), the effect of the interaction between cavity age and distance to forest edge was significant, while the effect of microtine index and the effects of the interactions between microtine index and the other variables were not significant (Table 1c).

### 3.2 Effect of year‐to‐year change in microtine rodent spring abundance

Among the models including the year‐to‐year change in microtine rodent trapping index, cavity age, and distance to forest edge, the most parsimonious, which also was the model with the highest AIC_c_ weight, included only the interaction between cavity age and distance to forest edge (Table S4). The effect of this interaction was significant (Table 2a). A model also including the year‐to‐year change in microtine index had the second highest rank and AIC_c_ weight (Table S4; ER = 0.46 to the most parsimonious model). In this model, the effect of the year‐to‐year change in microtine index was not significant, while the effect of the interaction between cavity age and distance to forest edge was significant (Table 2b). The non‐significant effect of the change in microtine index was positive, thus opposite to what was predicted. In the full model, which had a low AIC_c_ weight (ER < 0.10 to the most parsimonious model), the effect of distance to forest edge was significant, while the effects of year‐to‐year change in microtine index and its interaction with the other variables were not significant (Table 2c).

**TABLE 2:** Parameter estimates for a subset of GLMM models for the probability of predation of a goldeneye nest situated <40 km from the microtine rodent trapping site in a year when microtine rodents were trapped in relation to the year‐to‐year change in the microtine rodent spring trapping index, corrected for the random effect of nest box ID and temporal autocorrelation of year (*n* = 154 nests, 60 boxes, 33 years).

Explanatory variable	Estimate ± SE	*z*	*p*
*a*			
Intercept	−0.436 ± 0.228	−1.914	.056
Cavity age	0.274 ± 0.225	1.216	.22
Distance edge	−0.560 ± 0.264	−2.125	.034
Cavity age * Distance edge	−0.585 ± 0.256	−2.287	.022
*b*			
Intercept	−0.439 ± 0.226	−1.944	.052
Microtines change	0.169 ± 0.208	0.814	.42
Cavity age	0.285 ± 0.225	1.263	.21
Distance edge	−0.582 ± 0.265	−2.193	.028
Cavity age * Distance edge	−0.591 ± 0.257	−2.303	.021
*c*			
Intercept	−0.492 ± 0.253	−1.940	.052
Microtines change	0.116 ± 0.261	0.445	.66
Cavity age	0.304 ± 0.259	1.176	.24
Distance edge	−0.709 ± 0.317	−2.238	.025
Cavity age * Distance edge	−0.575 ± 0.311	−1.850	.064
Microtines change * Cavity age	0.089 ± 0.255	0.348	.73
Microtines change * Distance edge	0.456 ± 0.359	1.269	.20
Microtines change * Cavity age * Distance edge	−0.183 ± 0.353	−0.517	.61

*Note*: (a) The most parsimonious and highest‐ranked model (ΔAIC = 0.00). (b) The second most parsimonious and second highest‐ranked model (ΔAIC = 1.56). (c) The full model (ΔAIC >5.00). All models included in this analysis are described in Table S4. Generalized linear mixed‐effect models with log link function, binomial distribution, and Laplace approximation to the likelihood. Continuous explanatory variables are standardized.

### 3.3 Effects of proxies for microtine rodent abundance: All goldeneye nests

Based on the finding that boreal owl breeding occurrence and average clutch size within 40 km of each goldeneye nest, as well as the annual change in these values, substituted well for microtine rodent trapping index in explaining the probability of nest predation for goldeneye nests within 40 km from the microtine rodent trapping site (Appendix S2), we used these as proxies for microtine rodent abundance for all goldeneye nests in our study. In all four cases, the most parsimonious model, which also was the model with the highest weight, included only cavity age (Tables S9–S12). The probability of nest predation increased significantly with cavity age (Table 3).

**TABLE 3:** Parameter estimates from the highest‐ranked GLMM model among models that included the proxy for microtine rodent abundance for the probability of predation of a goldeneye nest situated anywhere in the study area, corrected for the random effect of nest box ID and temporal autocorrelation of year.

Explanatory variable	Estimate ± SE	*z*	*p*
*a*			
Intercept	−0.826 ± 0.275	−2.999	.0027
Owl breeding	0.151 ± 0.287	0.526	.60
Cavity age	0.420 ± 0.149	2.816	.0049
*b*			
Intercept	−0.804 ± 0.176	−4.579	<.0001
Change in owl breeding			
(L)	0.096 ± 0.265	0.362	.72
(Q)	−0.273 ± 0.205	−1.329	.18
Cavity age	0.423 ± 0.149	2.839	.0045
*c*			
Intercept	−0.683 ± 0.151	−4.535	<.0001
Owl clutch size	0.168 ± 0.125	1.344	.18
Cavity age	0.446 ± 0.149	2.994	.0028
*d*			
Intercept	−0.703 ± 0.147	−4.789	<.0001
Change owl clutch size	0.124 ± 0.123	1.009	.31
Cavity age	0.438 ± 0.137	3.194	.0014

*Note*: (a) Boreal owl breeding index (*n* = 509 nests, 181 boxes, 47 years, ΔAIC = 1.77). The model included in this analysis is described in Table S9. (b) Change in boreal owl breeding index (*n* = 508 nests, 181 boxes, 47 years, ΔAIC = 2.17). The model included in this analysis is described in Table S10. (c) Boreal owl clutch size (*n* = 445 nests, 176 boxes, 44 years, ΔAIC = 0.23). The model included in this analysis is described in Table S11. (d) Change in boreal owl clutch size (*n* = 383 nests, 167 boxes, 40 years, ΔAIC = 1.04). The model included in this analysis is described in Table S12. Generalized linear mixed‐effect models with log link function, binomial distribution, and Laplace approximation to the likelihood. Continuous explanatory variables are standardized. For the ordered factor “Change owl breeding,” “L” denotes the linear term, evaluating a straight‐line relationship, while “Q” denotes the quadratic term, capturing non‐linear patterns.

When the proxy for microtine abundance was boreal owl breeding occurrence, the highest‐ranked and most parsimonious among models including this variable also included cavity age and was the model with the fourth highest weight (Table S9, ER = 0.41). The effect of boreal owl breeding occurrence was not significant, while the effect of cavity age was significant (Table 3a). The non‐significant effect of boreal owl breeding occurrence was positive, thus opposite to what was predicted.

When the proxy for year‐to‐year change in microtine abundance was a year‐to‐year change in boreal owl breeding occurrence, the highest‐ranked and most parsimonious among models including this variable also included cavity age, and was the model with the fourth highest weight (Table S10, ER = 0.34). The effect of year‐to‐year change in boreal owl breeding occurrence was not significant, while the effect of cavity age was significant (Table 3b). The non‐significant effect of year‐to‐year change in boreal owl breeding occurrence was positive, thus opposite to what was predicted.

When the proxy based on whether boreal owls were found nesting or not within 40 km remained unchanged from the previous year, both years could be years without boreal owls nesting or both could be years with boreal owls nesting, that is, either two low microtine years in a row or two high microtine years in a row. The probability of predation of goldeneye nests was marginally non‐significantly higher in the former case than in the latter (0.57 (*n* = 14) vs. 0.34 (*n* = 300), *χ* ^2^ = 3.05, df = 1, *p* = .081). However, cavity age was significantly higher in the former case than in the latter (median = 14.5 (*n* = 14) vs. 5 (*n* = 300), Wilcoxon test, *χ* ^2^ = 15.68, *p* < .0001). When the two variables were entered into a model with probability of predation as response, the effect of 2 years in a row without vs. with boreal owl nesting <40 km away became non‐significant (*χ* ^2^ = 0.66, df = 1, *p* = .41), while the effect of cavity age remained significant (*χ* ^2^ = 6.55, df = 1, *p* = .011). Hence, the higher probability of nest predation in the second of two ensuing years with low microtine abundance than in the second of 2 years with high microtine abundance was due to goldeneyes having used older nest cavities in the former cases.

When the proxy for microtine abundance was boreal owl clutch size, the highest‐ranked and most parsimonious among models including this variable also included cavity age, and was the model with the second‐highest weight (Table S11). The effect of boreal owl clutch size was not significant, while the effect of cavity age was significant (Table 3c). The effect of boreal owl clutch size was positive, thus opposite to what was predicted.

When the proxy for year‐to‐year change in microtine abundance was a year‐to‐year change in boreal owl clutch size, the highest‐ranked and most parsimonious model including this variable also included cavity age, and was the model with the second‐highest weight (Table S12). The effect of year‐to‐year change in boreal owl clutch size was not significant, while the effect of cavity age was significant (Table 3d). The non‐significant effect of year‐to‐year change in boreal owl clutch size was positive, thus opposite to what was predicted.

**FIGURE 4:**
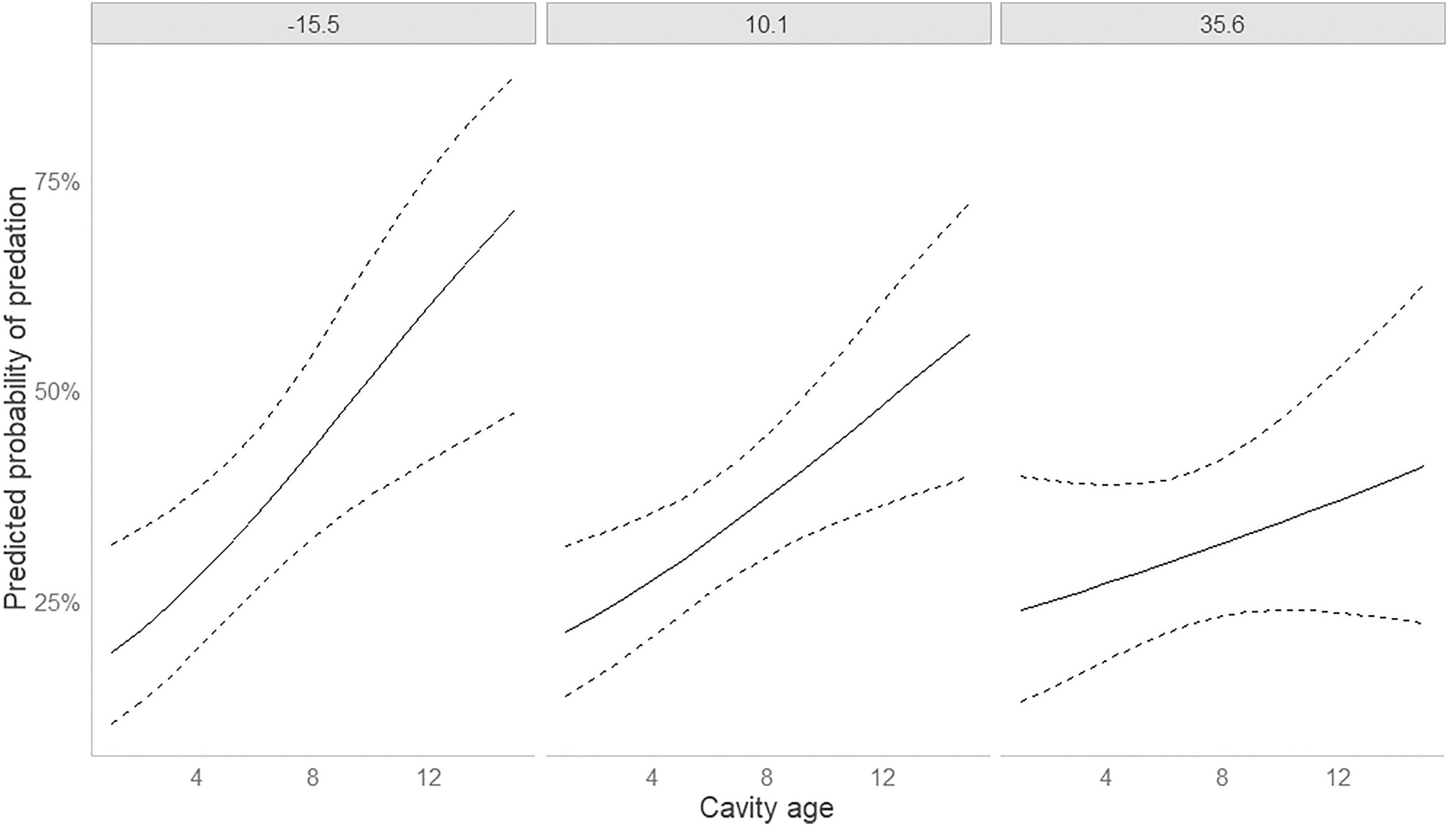
Predicted probability of predation of a goldeneye nest as function of cavity age for three values of distance from forest edge for all nests located anywhere in the study area (*n* = 509). The three values of distance from forest edge are the mean ± 1 SD (−15.5, 10.1, and 35.6 m).

When all boxes were included, the highest‐ranked model included only cavity age (Table S9), and the effect of cavity age was highly significant (Table 4a). The second highest‐ranked model included the interaction between cavity age and distance from forest edge (Table S9), but this interaction was not significant (Table 4b). The third highest‐ranked model included cavity age and distance from forest edge (Table S9), but the effect of distance from forest edge was not significant (Table 4c). Thus, the probability of nest predation increased with cavity age independent of distance from forest edge (Figure 4).

**TABLE 4:** Parameter estimates from the three highest‐ranked GLMM models for the probability of predation of a goldeneye nest situated anywhere in the study area, corrected for the random effect of nest box ID, and temporal autocorrelation of year (*n* = 509 nests, 181 boxes, 47 years).

Explanatory variable	Estimate ± SE	*z*	*p*
*a*			
Intercept	−0.707 ± 0.155	−4.559	<.0001
Cavity age	0.408 ± 0.147	2.774	.0055
*b*			
Intercept	−0.677 ± 0.153	−4.415	<.0001
Cavity age	0.431 ± 0.148	2.909	.0036
Distance to forest edge	−0.167 ± 0.153	−1.091	.27
Cavity age * Distance to forest edge	−0.215 ± 0.132	−1.627	.10
*c*			
Intercept	−0.711 ± 0.154	−4.607	<.0001
Cavity age	0.427 ± 0.149	2.875	.0040
Distance to forest edge	−0.163 ± 0.152	−1.073	.28

*Note*: (a) The highest‐ranked model (ΔAIC = 0.00). (b) The second highest‐ranked model (ΔAIC = 0.20). (c) The third highest‐ranked model (ΔAIC = 0.89). The models included in this analysis are described in Table S9. Generalized linear mixed‐effect models with log link function, binomial distribution, and Laplace approximation to the likelihood. Continuous explanatory variables are standardized.

## 4 DISCUSSION

We did not find any support for the APH in our data on pine marten predation of goldeneye nests. First, the non‐significant effect of the microtine rodent index on the probability of nest predation was positive, and thus opposite to that predicted by the APH. Second, the effect of a year‐to‐year change in microtine abundance on the probability of nest predation was non‐significant and positive, again opposite to the prediction of the APH. Third, as indirect measures of the microtine abundance at the time of the nesting of a goldeneye, we used the breeding occurrence and average clutch size of boreal owls within 40 km of each goldeneye nest. After having confirmed that these proxies, as well as their year‐to‐year change, had similar non‐significant and positive effects as the corresponding microtine indices for the probability of predation of goldeneye nests within 40 km from the microtine rodent trapping site, we were able to utilize a dataset with three times as many goldeneye nests, This covered a larger area, that is, more than 40 km from the microtine rodent trapping site (see Figure 1), and more years, that is, years when trapping of microtine rodents were not performed (1972–76 and 1979–80). However, neither in this extended dataset did the proxies for microtine abundance affect pine marten predation of goldeneye nests as predicted by the APH.

The microtine trapping index, as well as the proxies for microtine abundance were, if anything, positively related to the probability of predation. This indicates a higher probability of predation with higher microtine abundance in spring, which is opposite to the prediction by the APH. Similarly, the probability of pine marten predation of boreal owl nests in the same area tended to increase with increasing microtine rodent spring abundance (Sonerud, 2022). Pöysä et al. (2016) found a corresponding trend for pine marten predation of goldeneye nests in Finland and attributed it to a higher abundance of juvenile pine martens due to a higher survival during winters preceding a spring with high microtine abundance. These trends from the boreal forest fit with the fact that in the temperate forest in Poland, pine marten population density was strongly and positively correlated with the abundance of forest rodents (bank vole and yellow‐necked mouse (*Apodemus flavicolli*s) in the previous fall; Zalewski & Jedrzejewski, 2006).

The boreal owl breeding occurrence and average clutch size on which we based our indirect estimates of the microtine rodent abundance at each goldeneye nest were restricted to an area within 40 km from the actual goldeneye nest. This ensured that the estimate was spatially precise, because even in the years with low spatial synchrony in bank vole populations in our study area (1990–2003; Selås et al., 2021), bank vole populations <30–40 km apart exhibited statistically significant synchrony in growth patterns (Steen et al., 1996). Also, we regard our indirect estimates of the microtine abundance to be temporally precise, reflecting the abundance of microtines when the goldeneyes were nesting. On average, the boreal owls started egg laying earlier than sympatric goldeneyes, particularly in years with high microtine abundance. However, unless the microtine population underwent a crash after the boreal owls have laid their eggs, but before the goldeneye eggs had hatched, the estimate of microtine abundance taken as the boreal owl clutch size would be quite precise.

Based on the strong numerical response of the boreal owl to microtine rodents in Fennoscandia (Hörnfeldt et al., 2005; Korpimäki & Hakkarainen, 1991; Sonerud, 2022; Zarybnicka et al., 2015), we used two derived proxies for microtine abundance in the goldeneye breeding season. The simplest was the absence or presence of nesting boreal owls in a specified area, in our case within 40 km of each goldeneye nests. The more refined proxy was the average clutch size of boreal owls nesting within this area. Both proxies performed well, in the sense that their effect on pine marten predation of goldeneye nests was similar to the effect of microtine rodent trapping index, that is, non‐significant and positive. Data on breeding occurrence of boreal owls are easier to obtain than data on clutch size, as, for instance, observations of boreal owls nesting in woodpecker cavities, where clutch size is more demanding to record than in nest boxes, can be used, as well as observations of fledged broods of the boreal owl.

In the models with data from all study years and the whole study area, the variable explaining most of the variation in the probability of predation of a goldeneye nest was cavity age, with a positive effect. This confirms the pattern of increasing probability of predation with cavity age found for boreal owls, which was explained as individual pine martens finding more boxes as the years pass by, memorizing the spatial position of the nest boxes they have found, even boxes being empty when found, and revisiting them in later breeding seasons (Sonerud, 1985a, 2022). It also supports the suggestion by Elmberg and Pöysä (2011) and Pöysä et al. (2016) that predation of goldeneye nests by pine marten to a large extent is learned. Pine martens are relatively long‐lived. In Sweden, 51% of trapped individuals were older than 1 year, 29% were older than 2 years, and the oldest were 9 years (Helldin, 1999). In Poland, an estimated 9% of pine martens radio‐collared as subadult or adult were alive 5 years after (Zalewski & Jedrzejewski, 2006). Similar age distributions of trapped pine martens were found in older studies from Eastern Europe (see Sonerud, 1985a). Whether offspring may learn the location of tree cavities from their mother is unknown. Of four radio‐collared subadult males in a dense pine marten population in Poland, where average size of pine marten home range was less than half of that in Norway and Sweden (Zalewski & Jedrzejewski, 2006), two dispersed out of the natal area in spring, and two remained in the natal area, gradually expanding their range the next years (Zalewski, 2012).

In the models with data restricted to goldeneye nests in the area within 40 km from the microtine rodent trapping site, and to nests in years when trapping was conducted, amounting to around 30% of the goldeneye nests in our study, the variable explaining most of the variation in the probability of nest predation was distance to forest edge (measured as negative values for boxes in the forest interior and positive values for boxes in open habitats), either alone or in interaction with cavity age. The negative interaction between cavity age and distance to forest edge means that the increase in the probability of predation with increasing time elapsed since the box was installed became slower from the interior of forest stands 100 m from forest edge to the same distance from forest edge into open habitats. Because the pine marten favors habitats with a forest canopy and avoids open habitats (Brainerd & Rolstad, 2002), it would probably find boxes within a forest stand or at its edge sooner than boxes in open habitats, and would possibly also revisit them more often (cf. Sonerud, 1985a). Similarly, for the black woodpecker, the probability of pine marten nest predation was higher in mature forest than in clear‐cuts (Rolstad et al., 2000). Studies based on radio telemetry in boreal forests in Norway and Sweden found that pine martens prefer habitats with forest canopy and tall trees, and avoid clear‐cuts and other open areas, although they are able to utilize a wide range of succession stages of the forest (Brainerd & Rolstad, 2002). A recent study based on camera trapping in Norway found that pine marten occurred more often when old forest was present within 100 m of the camera trap, but not less often when clear‐cuts were present within 100 m (Angoh et al., 2023). The most important predator on the pine marten is the red fox (Lindström et al., 1995), which in the boreal forest, although being a habitat generalist, prefers clear‐cuts and other open areas (Storch et al., 1990). Thus, in our study the characteristics of a goldeneye nest site, taken as the age of a cavity and its distance from habitat with forest canopy, was more important for the probability of nest predation than the short‐term variation in prey availability for the pine marten, that is, microtine abundance.

## 5 CONCLUSION

Our data on predation of goldeneye nests by the pine marten did not support the APH hypothesis. In the complete dataset, the best predictor of the probability of predation of a goldeneye nest was cavity age, suggesting that the pine martens visited nest boxes they had learned no matter the microtine abundance. This confirms the results from a corresponding study of pine marten predation of boreal owl nests (Sonerud, 2022). In the latter, however, data from years with very low microtine abundance (“crash years”) were very scarce due to the strong numerical response of boreal owls to microtine abundance. Therefore, it is noteworthy that the present study, with data on a bird nesting independent of microtine abundance, ends up with the same main result. Although these two studies were based on overlapping nest box populations in overlapping study areas and were non‐experimental, previous experimental studies based on relocation of nest boxes from the same nest box population have demonstrated that spatial memory is important in pine marten predation of nests in tree cavities (Sonerud, 1989, 1993).

## AUTHOR CONTRIBUTIONS

**Geir A. Sonerud:** Conceptualization (lead); data curation (lead); formal analysis (equal); funding acquisition (equal); investigation (equal); methodology (equal); project administration (equal); resources (equal); software (supporting); supervision (equal); validation (lead); visualization (equal); writing – original draft (lead); writing – review and editing (lead). **Helge E. Grønlien:** Conceptualization (supporting); data curation (supporting); formal analysis (supporting); funding acquisition (equal); investigation (equal); methodology (equal); project administration (equal); resources (equal); software (supporting); supervision (equal); validation (supporting); visualization (supporting); writing – original draft (supporting); writing – review and editing (supporting). **Ronny Steen:** Conceptualization (supporting); data curation (supporting); formal analysis (equal); funding acquisition (supporting); investigation (supporting); methodology (equal); project administration (supporting); resources (equal); software (lead); supervision (supporting); validation (supporting); visualization (equal); writing – original draft (supporting); writing – review and editing (supporting).

## FUNDING INFORMATION

This study received funding from the Nansen Endowment (grant numbers 102/87, 101/88, 111/89, 82/90, 97/91, 101/92, 203/93, 197b/94, and 87/95); Norwegian Environment Agency; and Lillehammer Municipality.

## CONFLICT OF INTEREST STATEMENT

The authors declare that they have no competing interests.

## Supporting information


Appendices S1 and S2

## Data Availability

Relevant data files and scripts are preliminarily archived as a private link in the FigShare Repository: https://figshare.com/s/837855fb030413073bc3.
