# Supplementary material for: Pine marten predation of common goldeneye nests: Effects of cavity age and habitat override any effect of microtine rodent abundance
Source: Ecol Evol. 2023 Oct 24;13(10):e10643. doi: 10.1002/ece3.10643 (PMC10594075; doi:10.1002/ece3.10643)
Supplement: Supplementary file 1 — Appendices S1 and S2 [file ECE3-13-e10643-s001.pdf]

Supporting information to:

Pine marten predation of goldeneye nests: effects of cavity age and habitat override any effect of microtine rodent abundance

Geir A. Sonerud [1], Helge E. Grønlien [2] and Ronny Steen [1]

Ecology and Evolution, 2023

Article Doi: 10.1002/ece3.10643

Appendix S1: Tables S1 – S12. ………………………………………………… p. 2

Appendix S2: Effects of boreal owl nest occurrence and clutch size as proxies for microtine rodent abundance: goldeneye nests within 40 km from the microtine rodent trapping site. ..……….…………………………………… p. 14

[1] Faculty of Environmental Sciences and Natural Resource Management, Norwegian University of Life Sciences, P. O. Box 5003, NO-1432 Ås, Norway

[2] Anders Bjørnsgårds vei 7, NO-2625 Fåberg, Norway

Corresponding author: e-mail: geir.sonerud@nmbu.no

Table S1 Number of goldeneye nests distributed on number of nest boxes used.

	All nests (1972 - 2020)		Nests < 40 km from trapping site in trapping years (1977-78 and 1981-2020)	
Nests per box	Boxes	Nests	Boxes	Nests
1	65	65	24	24
2	47	94	19	38
3	20	60	5	15
4	14	56	6	24
5	7	35	1	5
6	13	78	3	18
7	4	28	0	0
8	9	72	4	32
10	1	10	0	0
11	1	11	0	0
Total	181	509	62	156

Table S2 Number of goldeneye nests distributed on number of years.

	All nests (1972 - 2020)		Nests < 40 km from trapping site in trapping years (1977-78 and 1981-2020)	
Nests per year	Years	Nests	Years	Nests
1	5	5	2	2
2	6	12	8	16
3	2	6	6	18
4	7	28	5	20
5	3	15	3	15
6	1	6	2	12
7	1	7	0	0
8	1	8	4	32
9	2	18	1	9
10	0	0	1	10
11	0	0	2	22
13	1	13	0	0
14	2	28	0	0
16	1	16	0	0
17	1	17	0	0
18	1	18	0	0
19	1	19	0	0
20	2	40	0	0
21	1	21	0	0
22	2	44	0	0
23	1	23	0	0
24	1	24	0	0
26	1	26	0	0
27	1	27	0	0
28	1	28	0	0
29	1	29	0	0
31	1	31	0	0
Total	47	509	34	156

Table S3 Model selection based on Akaike´s information criterion (AIC) to determine which variables influenced the probability of predation of a goldeneye nest situated < 40 km from the microtine rodent trapping site in a year when microtine rodents were trapped (*n* = 156), corrected for the random effect of nest box ID (n = 62) and temporal autocorrelation of year (*n* = 34). MicRod (range 0.10 – 8.04) denotes the microtine rodent spring trapping index (see text for details). Cav (range 1 – 15) denotes the time since the actual nest box was installed (see text for details). Dist (range -100 – 100) denotes the shortest distance from the nest box to the nearest edge between forest and an open habitat (see text for details). The GLMM models are sorted by their degree of support from the data, as indicated by the AICc weight (w). Models with ΔAIC ≤ 2.0 from the best model are shown in bold.

Variables	LogLikelihood	df	AICc	ΔAIC	AIC w
**Cav + Dist + MicRod + Cav*Dist**	**-94.20**	**8**	**205.38**	**0.00**	**0.291**
**Cav + Dist + Cav*Dist**	**-95.50**	**7**	**205.76**	**0.38**	**0.240**
**Cav + Dist + MicRod + Cav*Dist + Cav*MicRod**	**-93.77**	**9**	**206.76**	**1.38**	**0.146**
Cav + Dist + MicRod + Cav*Dist + Dist*MicRod	-94.20	9	207.63	2.25	0.094
Dist + MicRod	-98.13	6	208.83	3.45	0.052
Cav + Dist + MicRod + Cav*Dist + Cav*MicRod + Dist*MicRod	-93.76	10	209.04	3.66	0.047
Cav + Dist + MicRod	-97.16	7	209.08	3.70	0.046
Cav + Dist	-98.50	6	209.57	4.19	0.036
Dist	-99.94	5	210.29	4.91	0.025
Cav + Dist + MicRod + Cav*Dist + Cav*MicRod + Dist*MicRod + Cav*Dist*MicRod	-93.25	11	210.33	4.95	0.024

Table S4 Model selection based on Akaike´s information criterion (AIC) to determine which variables influenced the probability of predation of a goldeneye nest situated < 40 km from the microtine rodent trapping site in a year when microtine rodents had also been trapped in the previous year (*n* = 154), corrected for the random effect of nest box ID (n = 60) and temporal autocorrelation of year (*n* = 33). MicRodCh (range -3.53 – 5.03) denotes the change in the microtine rodent trapping index from the previous spring to the current spring (see text for details). Cav (range 1 – 15) denotes the time since the actual nest box was installed (see text for details). Dist (range -100 – 100) denotes the shortest distance from the nest box to the nearest edge between forest and an open habitat (see text for details). The GLMM models are sorted by their degree of support from the data, as indicated by the AICc weight (w). Models with ΔAIC ≤ 2.0 from the best model are shown in bold.

Variables	LogLikelihood	df	AICc	ΔAIC	AIC w
**Cav + Dist + Cav*Dist**	**-93.64**	**7**	**202.04**	**0.00**	**0.438**
**Cav + Dist + MicRodCh + Cav*Dist**	**-93.30**	**8**	**203.60**	**1.56**	**0.202**
Cav + Dist + MicRodCh + Cav*Dist + Dist*MicRodCh	-92.56	9	204.37	2.33	0.138
Cav + Dist + MicRodCh + Cav*Dist + Cav*MicRodCh	-93.30	9	205.85	3.81	0.066
Cav + Dist	-96.67	6	205.90	3.86	0.064
Cav + Dist + MicRodCh + Cav*Dist + Cav*MicRodCh + Dist*MicRodCh	-92.48	10	206.50	4.46	0.047
Dist	-98.07	5	206.55	4.51	0.046

Table S5 Model selection based on Akaike´s information criterion for small sample size (AICc) to determine which variables influenced the probability of predation of a goldeneye nest (*n* = 156) situated < 40 km from the microtine rodent trapping site in a year when microtine rodents were trapped (1977-78 and 1981-2020), corrected for the random effect of nest box ID (n = 62) and temporal autocorrelation of year (*n* = 34). OwlBr (1 or 0) denotes whether boreal owl nests were found breeding or not in boxes < 40 km from the actual goldeneye nest in the actual year (see text for details). Cav (range 1 – 15) denotes the time since the actual nest box was installed, with 1 denoting the first nesting season the box was available (see text). Dist (range -100 – 100) denotes the shortest distance from the nest box to the nearest edge between forest and an open habitat, with zero for boxes situated at the edge between forest and open habitat, a negative value for boxes situated within a forest stand, and a positive value for boxes situated in a tree in a clear-cut or a bog (see text for details). The GLMM models are sorted by their degree of support from the data, as indicated by the AICc weight (w). Models with ΔAICc ≤ 2.0 from the best model are shown in bold.

Variables	LogLikelihood	df	AICc	ΔAICc	AICc w
**Cav + Dist + OwlBr + Cav*Dist + Dist*OwlBr**	**-93.20**	**9**	**205.62**	**0.00**	**0.255**
**Cav + Dist + Cav*Dist**	**-95.50**	**7**	**205.76**	**0.14**	**0.238**
**Cav + Dist + OwlBr + Cav*Dist + Cav*OwlBr + Dist*OwlBr**	**-92.59**	**10**	**206.69**	**1.07**	**0.150**
**Cav + Dist + OwlBr + Cav*Dist**	**-95.27**	**8**	**207.51**	**1.89**	**0.099**
Cav + Dist + OwlBr + Cav*Dist + Cav*OwlBr + Dist*OwlBr + Cav*Dist*OwlBr	-92.26	11	208.35	2.73	0.065
Cav + Dist + OwlBr + Cav*Dist + Cav*OwlBr	-94.85	9	208.94	3.32	0.049
Cav + Dist + OwlBr + Dist*OwlBr	-95.99	8	208.96	3.34	0.048
Cav + Dist	-98.50	6	209.57	3.95	0.036
Dist + OwlBr + Dist*OwlBr	-97.43	7	209.62	4.00	0.035
Dist	-99.94	5	210.29	4.67	0.025

Table S6 Model selection based on Akaike´s information criterion for small sample size (AICc) to determine which variables influenced the probability of predation of a goldeneye nest (*n* = 156) situated < 40 km from the microtine rodent trapping site in a year when microtine rodents were trapped (1977-78 and 1981-2020), corrected for the random effect of nest box ID (n = 62) and temporal autocorrelation of year (*n* = 34). OwlBrCh (-1 or 0 or 1) denotes the change from the previous year in whether boreal owl nests were found breeding or not in boxes < 40 km from the actual goldeneye nest (see text for details). Cav (range 1 – 15) denotes the time since the actual nest box was installed, with 1 denoting the first nesting season the box was available (see text). Dist (range -100 – 100) denotes the shortest distance from the nest box to the nearest edge between forest and an open habitat, with zero for boxes situated at the edge between forest and open habitat, a negative value for boxes situated within a forest stand, and a positive value for boxes situated in a tree in a clear-cut or a bog (see text for details). The GLMM models are sorted by their degree of support from the data, as indicated by the AICc weight (w). Models with ΔAICc ≤ 2.0 from the best model are shown in bold.

Variables	LogLikelihood	df	AICc	ΔAICc	AICc w
**Cav + Dist + Cav*Dist**	**-95.50**	**7**	**205.76**	**0.00**	**0.477**
Cav + Dist + OwlBrCh + Cav*Dist + Dist*OwlBrCh	-91.97	11	207.77	2.01	0.174
Cav + Dist + OwlBrCh + Cav*Dist + Cav*OwlBrCh + Dist*OwlBrCh	-90.04	13	208.65	2.89	0.113
Cav + Dist	-98.50	6	209.57	3.81	0.071
Cav + Dist + OwlBrCh + Cav*Dist	-95.19	9	209.61	3.85	0.070
Dist	-99.94	5	210.29	4.53	0.050
Cav + Dist + OwlBrCh + Cav*Dist + Cav*OwlBrCh	-93.31	11	210.46	4.70	0.046

Table S7 Model selection based on Akaike´s information criterion for small sample size (AICc) to determine which variables influenced the probability of predation of a goldeneye nest (*n* = 132) situated < 40 km from the microtine rodent trapping site in a year when microtine rodents were trapped (1977-78 and 1981-2020), corrected for the random effect of nest box ID (n = 60) and temporal autocorrelation of year (*n* = 31). Clutch (range 0.0 – 6.5) denotes the average clutch size of boreal owl nests situated < 40 km from the actual goldeneye nest (see text for details). Cav (range 1 – 15) denotes the time since the actual nest box was installed, with 1 denoting the first nesting season the box was available (see text). Dist (range -100 – 100) denotes the shortest distance from the nest box to the nearest edge between forest and an open habitat, with zero for boxes situated at the edge between forest and open habitat, a negative value for boxes situated within a forest stand, and a positive value for boxes situated in a tree in a clear-cut or a bog (see text for details). The GLMM models are sorted by their degree of support from the data, as indicated by the AICc weight (w). Models with ΔAICc ≤ 2.0 from the best model are shown in bold.

Variables	LogLikelihood	df	AICc	ΔAICc	AICc w
**Cav + Dist + Clutch + Cav*Dist + Cav*Clutch + Dist*Clutch**	**-78.82**	**10**	**179.47**	**0.00**	**-**
**Cav + Dist + Clutch + Cav*Dist + Dist*Clutch**	**-80.28**	**9**	**180.03**	**0.56**	**-**
**Cav + Dist + Cav*Dist**	**-82.65**	**7**	**180.21**	**0.74**	**-**
**Cav + Dist + Clutch + Dist*Clutch**	**-82.15**	**8**	**181.46**	**1.99**	**-**
Cav + Dist + Clutch + Cav*Clutch + Dist*Clutch	-81.12	9	181.72	2.25	-
Cav + Dist + Clutch + Cav*Dist + Cav*Clutch + Dist*Clutch + Cav*Dist*Clutch	-78.82	11	181.83	2.36	-
Cav + Dist + Clutch + Cav*Dist	-82.60	8	182.36	2.89	-
Cav + Dist + Clutch + Cav*Dist + Cav*Clutch	-81.46	9	182.40	2.93	-
Dist + Clutch + Dist*Clutch	-84.02	7	182.94	3.47	-
Cav + Dist	-85.33	6	183.33	3.86	-
Dist	-86.89	5	184.25	4.78	-

Table S8 Model selection based on Akaike´s information criterion for small sample size (AICc) to determine which variables influenced the probability of predation of a goldeneye nest (*n* = 106) situated < 40 km from the microtine rodent trapping site in a year when microtine rodents were trapped (1977-78 and 1981-2020), corrected for the random effect of nest box ID (n = 55) and temporal autocorrelation of year (*n* = 27). ClutchCh (range -6.5 – 6.5) denotes the change in average clutch size of boreal owl nests situated < 40 km from the actual goldeneye nest (see text for details). Cav (range 1 – 15) denotes the time since the actual nest box was installed, with 1 denoting the first nesting season the box was available (see text). Dist (range -100 – 100) denotes the shortest distance from the nest box to the nearest edge between forest and an open habitat, with zero for boxes situated at the edge between forest and open habitat, a negative value for boxes situated within a forest stand, and a positive value for boxes situated in a tree in a clear-cut or a bog (see text for details). The GLMM models are sorted by their degree of support from the data, as indicated by the AICc weight (w). Models with ΔAICc ≤ 2.0 from the best model are shown in bold.

Variables	LogLikelihood	df	AICc	ΔAICc	AICc w
**Cav**	**-69.07**	**5**	**148.74**	**0.00**	**-**
**Intercept**	**-70.78**	**4**	**149.96**	**1.22**	**-**
Cav + Dist	-69.02	6	150.89	2.15	-
Cav + ClutchCh	-69.03	6	150.91	2.17	-
Cav + Dist + Cav*Dist	-68.13	7	151.39	2.65	-
ClutchCh	-70.73	5	152.07	3.33	-
Cav + ClutchCh + Cav*ClutchCh	-68.94	7	153.03	4.29	-
Cav + Dist + ClutchCh	-68.98	7	153.10	4.36	-
Cav + Dist + ClutchCh + Cav*Dist	-68.08	8	153.64	4.90	-

Table S9 Model selection based on Akaike´s information criterion for small sample size (AICc) to determine which variables influenced the probability of predation of a goldeneye nest (*n* = 509), corrected for the random effect of nest box ID (n = 181) and temporal autocorrelation of year (*n* = 47). OwlBr (1 or 0) denotes whether boreal owl nests were found breeding or not in boxes < 40 km from the actual goldeneye nest in the actual year (see text for details). Cav (range 1 – 15) denotes the time since the actual nest box was installed, with 1 denoting the first nesting season the box was available (see text). Dist (range -100 – 100) denotes the shortest distance from the nest box to the nearest edge between forest and an open habitat, with zero for boxes situated at the edge between forest and open habitat, a negative value for boxes situated within a forest stand, and a positive value for boxes situated in a tree in a clear-cut or a bog (see text for details). The GLMM models are sorted by their degree of support from the data, as indicated by the AICc weight (w). Models with ΔAICc ≤ 2.0 from the best model are shown in bold.

Variables	LogLikelihood	df	AICc	ΔAICc	AICc w
**Cav**	**-308.17**	**5**	**626.46**	**0.00**	**0.221**
**Cav + Dist + Cav*Dist**	**-306.22**	**7**	**626.66**	**0.20**	**0.200**
**Cav + Dist**	**-307.59**	**6**	**627.36**	**0.89**	**0.141**
**Cav + OwlBr**	**-308.03**	**6**	**628.23**	**1.77**	**0.091**
Cav + Dist + OwlBr + Cav*Dist	-306.10	8	628.48	2.02	0.081
Cav + Dist + OwlBr + Cav*Dist + Cav*OwlBr + Dist*OwlBr + Cav*Dist*OwlBr	-303.23	11	628.99	2.53	0.062
Cav + Dist + OwlBr	-307.45	7	629.12	2.66	0.059
Cav + OwlBr + Cav*OwlBr	-308.01	7	630.24	3.78	0.033
Cav + Dist + OwlBr + Cav*Dist + Dist*OwlBr	-306.00	9	630.36	3.90	0.031
Cav + Dist + OwlBr + Cav*Dist + Cav*OwlBr	-306.04	9	630.44	3.98	0.030
Cav + Dist + OwlBr + Dist*OwlBr	-307.11	8	630.51	4.05	0.029
Cav + Dist + OwlBr + Cav*OwlBr	-307.43	8	631.14	4.68	0.021

Table S10 Model selection based on Akaike´s information criterion for small sample size (AICc) to determine which variables influenced the probability of predation of a goldeneye nest (*n* = 508), corrected for the random effect of nest box ID (n = 181) and temporal autocorrelation of year (*n* = 47). OwlBrCh (-1 or 0 or 1) denotes the change from the previous year in whether boreal owl nests were found breeding or not in boxes < 40 km from the actual goldeneye nest (see text for details). Cav (range 1 – 15) denotes the time since the actual nest box was installed, with 1 denoting the first nesting season the box was available (see text). Dist (range -100 – 100) denotes the shortest distance from the nest box to the nearest edge between forest and an open habitat, with zero for boxes situated at the edge between forest and open habitat, a negative value for boxes situated within a forest stand, and a positive value for boxes situated in a tree in a clear-cut or a bog (see text for details). The GLMM models are sorted by their degree of support from the data, as indicated by the AICc weight (w). Models with ΔAICc ≤ 2.0 from the best model are shown in bold.

Variables	LogLikelihood	df	AICc	ΔAICc	AICc w
**Cav**	**-307.80**	**5**	**625.72**	**0.00**	**0.253**
**Cav + Dist + Cav*Dist**	**-305.85**	**7**	**625.93**	**0.21**	**0.227**
**Cav + Dist**	**-307.23**	**6**	**626.63**	**0.91**	**0.160**
Cav + OwlBrCh	-306.83	7	627.89	2.17	0.085
Cav + Dist + OwlBrCh + Cav*Dist	-304.77	9	627.91	2.19	0.084
Cav + Dist + OwlBrCh + Cav*Dist + Dist*OwlBrCh	-302.76	11	628.06	2.34	0.078
Cav + Dist + OwlBrCh + Dist*OwlBrCh	-304.12	10	628.68	2.96	0.057
Cav + Dist + OwlBrCh	-306.25	8	628.79	3.07	0.054

Table S11 Model selection based on Akaike´s information criterion for small sample size (AICc) to determine which variables influenced the probability of predation of a goldeneye nest (*n* = 445), corrected for the random effect of nest box ID (n = 176) and temporal autocorrelation of year (*n* = 44). Clutch (range 0.0 – 7.0) denotes the average clutch size of boreal owl nests situated < 40 km from the actual goldeneye nest (see text for details). Cav (range 1 – 15) denotes the time since the actual nest box was installed, with 1 denoting the first nesting season the box was available (see text). Dist (range -100 – 100) denotes the shortest distance from the nest box to the nearest edge between forest and an open habitat, with zero for boxes situated at the edge between forest and open habitat, a negative value for boxes situated within a forest stand, and a positive value for boxes situated in a tree in a clear-cut or a bog (see text for details). The GLMM models are sorted by their degree of support from the data, as indicated by the AICc weight (w). Models with ΔAICc ≤ 2.0 from the best model are shown in bold.

Variables	LogLikelihood	df	AICc	ΔAICc	AICc w
**Cav**	**-273.57**	**5**	**557.27**	**0.00**	**-**
**Cav + Clutch**	**-272.66**	**6**	**557.50**	**0.23**	**-**
**Cav + Dist + Clutch + Dist*Clutch**	**-270.60**	**8**	**557.53**	**0.26**	**-**
**Cav + Clutch + Cav*Clutch**	**-272.01**	**7**	**558.27**	**1.00**	**-**
**Cav + Dist + Clutch + Cav*Dist + Cav*Clutch + Dist*Clutch + Cav*Dist*Clutch**	**-267.85**	**11**	**558.32**	**1.04**	**-**
**Cav + Dist + Clutch + Cav*Clutch + Dist*Clutch**	**-270.26**	**9**	**558.93**	**1.66**	**-**
**Cav + Dist + Clutch + Cav*Dist + Dist*Clutch**	**-270.33**	**9**	**559.08**	**1.81**	**-**
**Cav + Dist**	**-273.47**	**6**	**559.13**	**1.86**	**-**
**Cav + Dist + Cav*Dist**	**-272.44**	**7**	**559.14**	**1.87**	**-**
**Cav + Dist + Clutch**	**-272.56**	**7**	**559.38**	**2.11**	**-**
**Cav + Dist + Clutch + Cav*Dist**	**-271.67**	**8**	**559.66**	**2.39**	**-**
Cav + Dist + Clutch + Cav*Clutch	-271.93	8	560.19	2.92	-
Cav + Dist + Clutch + Cav*Dist + Cav*Clutch + Dist*Clutch	-270.02	10	560.54	3.27	-
Cav + Dist + Clutch + Cav*Dist + Age*Clutch	-271.20	9	560.81	3.54	-

Table S12 Model selection based on Akaike´s information criterion for small sample size (AICc) to determine which variables influenced the probability of predation of a goldeneye nest (*n* = 383), corrected for the random effect of nest box ID (n = 167) and temporal autocorrelation of year (*n* = 40). ClutchCh (range -6.5 – 7.0) denotes the change in average clutch size of boreal owl nests situated < 40 km from the actual goldeneye nest (see text for details). Cav (range 1 – 15) denotes the time since the actual nest box was installed, with 1 denoting the first nesting season the box was available (see text). Dist (range -100 – 100) denotes the shortest distance from the nest box to the nearest edge between forest and an open habitat, with zero for boxes situated at the edge between forest and open habitat, a negative value for boxes situated within a forest stand, and a positive value for boxes situated in a tree in a clear-cut or a bog (see text for details). The GLMM models are sorted by their degree of support from the data, as indicated by the AICc weight (w). Models with ΔAICc ≤ 2.0 from the best model are shown in bold.

Variables	LogLikelihood	df	AICc	ΔAICc	AICc w
**Cav**	**-234.93**	**5**	**480.01**	**0.00**	**-**
**Cav + ClutchCh**	**-234.42**	**6**	**481.05**	**1.04**	**-**
**Cav + Dist**	**-234.90**	**6**	**482.01**	**2.00**	**-**
Cav + ClutchCh + Cav*ClutchCh	-234.03	7	482.37	2.36	-

Effects of boreal owl nest occurrence and clutch size as proxies for microtine rodent abundance: goldeneye nests within 40 km from the microtine rodent trapping site

In order to test the APH also with data from goldeneye nests situated > 40 km from the microtine rodent trapping site, and with data from nests in years when microtine trapping was not conducted (1972-76 and 1979-80), we used boreal owl breeding occurrence and average boreal owl clutch size < 40 km of each goldeneye nest as proxies for microtine rodent abundance.

To see how representative these proxies were, we substituted microtine abundance with these proxies for goldeneye nests situated < 40 km from the trapping site in the years when trapping of microtine rodents was conducted. When the proxy for microtine abundance was boreal owl breeding occurrence, the most parsimonious model included only the interaction between cavity age and distance to forest edge (Table S5). This model had the second highest weight (ER = 0.93, Table S5). One of the models with boreal owl breeding occurrence included had the highest weight (Table S5). In this model, the effect of boreal owl breeding occurrence was not significant, while the effect of the interaction between cavity age and distance to forest edge was significant (Table S13a). The most parsimonious among the models with boreal owl breeding occurrence included had the fourth highest weight (ER = 0.39, Table S5). In this model, the effect of boreal owl breeding occurrence was not significant, while the effect of the interaction between cavity age and distance to forest edge was significant (Table S14a). The non-significant effect of boreal owl breeding occurrence was positive, which is opposite to what was predicted. Thus, the effect of boreal owl breeding occurrence was similar to the effect of microtine index (cf. Table 1b).

When the proxy for microtine abundance was year-to-year change in boreal owl breeding occurrence, the most parsimonious model, which also was the model with highest AICc weight, included only the interaction between cavity age and distance to forest edge (Table S6). The highest-ranked model among the models with year-to-year change in boreal owl breeding occurrence included had the second highest weight (ER = 0.36, Table S6). In this model, the effect of change in boreal owl breeding occurrence was not significant, while the effect of the interaction between cavity age and distance to forest edge was significant (Table S13b). The most parsimonious among the models with year-to-year change in boreal owl breeding occurrence included had the fifth

highest weight (ER = 0.15, Table S6). In this model, the effect of change in boreal owl breeding occurrence was not significant, while the effect of the interaction between cavity age and distance to forest edge was significant (Table S14b). The effect of year-to-year change in boreal owl breeding occurrence was positive, which is opposite to what was predicted. Thus, the effect of year-to-year change in boreal owl breeding occurrence was similar to the effect of year-to-year change in microtine index (cf. Table 2b).

When the proxy for microtine abundance was boreal owl clutch size, the most parsimonious model, which was the model with the third highest AICc weight, included only the interaction between cavity age and distance to forest edge (Table S7). One of the models with boreal owl clutch size included had the highest weight (Table S7). In this model, the effects of the two interactions involving boreal owl clutch size were marginally non-significant (Table S14c). The most parsimonious among models with boreal owl clutch size included had the fourth highest weight (Table S7). In this model, the effect of the interaction between boreal owl clutch size and distance to forest edge was significant (Table S14c). The positive interaction between boreal owl clutch size and distance to forest edge means that the probability of predation of a goldeneye nest as function of boreal owl clutch size increased more rapidly with increasing distance from forest interior into open habitats. The positive effects of boreal owl clutch size were opposite to what was predicted. Thus, the effect of boreal owl clutch size was similar to the effect of microtine index (cf. Table 1b).

Finally, when the proxy for microtine abundance was year-to-year change in boreal owl clutch size, the most parsimonious model, which was the model with second highest AICc weight, included only intercept (Table S8). The highest-ranked among the models with annual change in boreal owl clutch size included was the fourth highest-ranked (Table S8). In this model, the effect of year-to-year change in boreal owl clutch size was not significant (Table S13d). The most parsimonious among models with annual change in boreal owl clutch size included was the sixth highest-ranked (Table S8). In this model, the effect of year-to-year change in boreal owl clutch size was not significant (Table S14d). The positive effect of annual change in boreal owl clutch size in both models is opposite to what was predicted. Thus, the effect of annual change in boreal owl clutch size was similar to the effect of annual change in microtine index (cf. Table 2b).

TABLE S13 Parameter estimates from the highest-ranked GLMM model among models that included the proxy for microtine rodent abundance (boreal owl breeding index and boreal owl clutch size) for the probability of predation of a goldeneye nest situated < 40 km from the microtine rodent trapping site in a year when microtine rodents were trapped, corrected for the random effect of nest box ID and temporal autocorrelation of year.

Explanatory variable	Estimate ± SE	z	*P*
**a**			
Intercept	-1.389 ± 0.901	-1.542	0.12
Owl breeding	1.073 ± 0.918	1.169	0.24
Cavity age	0.304 ± 0.231	1.316	0.19
Distance to forest edge	-2.663 ± 1.538	-1.731	0.083
Cavity age * Distance to forest edge	-0.552 ± 0.257	-2.153	0.031
Owl breeding * Distance to forest edge	2.232 ± 1.532	1.457	0.15
**b**			
Intercept	-0.728 ± 0.388	-1.874	0.061
Change owl breeding			
(L)	0.658 ± 0.730	0.902	0.37
(Q)	-0.573 ± 0.496	-1.154	0.25
Cavity age	0.333 ± 0.254	1.313	0.19
Distance to forest edge	-0.980 ± 0.594	-1.650	0.099
Cavity age * Distance to forest edge	-0.650 ± 0.291	-2.235	0.025
Change owl breeding * Distance to forest edge	2.232 ± 1.532	1.457	0.15
(L)	2.163 ± 1.220	1.772	0.076
(Q)	-0.442 ± 0.717	-0.617	0.54
**c**			
Intercept	-0.448 ± 0.224	-2.002	0.045
Owl clutch size	0.100 ± 0.235	0.426	0.67
Cavity age	0.329 ± 0.228	1.445	0.15
Distance to forest edge	-0.575 ± 0.288	-1.981	0.048
Cavity age * Distance to forest edge	-0.495 ± 0.250	-1.828	0.068
Owl clutch size * Cavity age	0.422 ± 0.255	1.653	0.098

Owl clutch size * Distance to forest edge	0.691 ± 0.363	1.903	0.057
**d**			
Intercept	-0.436 ± 0.218	-2.005	0.045
Change in owl clutch size	0.058 ± 0.206	0.283	0.78
Cavity age	0.412 ± 0.235	1.757	0.079

a Boreal owl breeding index (Table S5, n = 156 nests, 62 boxes, 34 years, ΔAIC = -0.14). **b** Change in boreal owl breeding index from previous year (Table S6, n = 156 nests, 62 boxes, 34 years, ΔAIC = 2.01). **c** Boreal owl clutch size (Table S7, n = 132 nests, 60 boxes, 31 years, ΔAIC = -0.74). **d** Change in boreal owl clutch size from previous year (Table S8, n = 106 nests, 55 boxes, 27 years, ΔAIC = 2.17). ΔAIC denotes the difference in AIC from the most parsimonious model. All models included in this analysis are described in Tables S5-S8. Generalized linear mixed-effect models with log link function, binomial distribution, and Laplace approximation to the likelihood. Explanatory variables are standardized. For the ordered factor “Change owl breeding”, “L” denotes the linear term, evaluating a straight-line relationship, while “Q” denotes the quadratic term, capturing non-linear patterns.

TABLE S14 Parameter estimates from the most parsimonious GLMM model among models that included the proxy for microtine rodent abundance (boreal owl breeding index and boreal owl clutch size) for the probability of predation of a goldeneye nest situated < 40 km from the microtine rodent trapping site in a year when microtine rodents were trapped, corrected for the random effect of nest box ID and temporal autocorrelation of year.

Explanatory variable	Estimate ± SE	z	*P*
**a**			
Intercept	-0.687 ± 0.504	-1.363	0.17
Owl breeding	0.365 ± 0.535	0.682	0.50
Cavity age	0.298 ± 0.226	1.318	0.19
Distance to forest edge	-0.544 ± 0.260	-2.092	0.037
Cavity age * Distance to forest edge	-0.572 ± 0.251	-2.277	0.023
**b**			
Intercept	-0.687 ± 0.504	-1.363	0.17
Change in owl breeding			
(L)	0.151 ± 0.469	0.321	0.75
(Q)	-0.256 ± 0.354	-0.722	0.47
Cavity age	0.312 ± 0.230	1.355	0.18
Distance to forest edge	-0.542 ± 0.261	-2.077	0.038
Cavity age * Distance to forest edge	-0.573 ± 0.252	-2.275	0.023
**c**			
Intercept	-0.451 ± 0.219	-2.058	0.040
Owl clutch size	0.198 ± 0.234	0.846	0.40
Cavity age	0.399 ± 0.211	1.894	0.058
Distance to forest edge	-0.566 ± 0.271	-2.087	0.037
Owl clutch size * Distance to forest edge	0.714 ± 0.353	2.024	0.043
**d**			
Intercept	-0.429 ± 0.256	-1.901	0.057
Change in owl clutch size	0.065 ± 0.211	0.307	0.76

a Boreal owl breeding index (Table S5, n = 156 nests, 62 boxes, 34 years, ΔAIC = 1.89). **b** Change in boreal owl breeding index from previous year (Table S6, n = 156 nests, 62 boxes, 34 years, ΔAIC = 1.84). **c** Boreal owl clutch size (Table S7, n = 132 nests, 60 boxes, 31 years, ΔAIC = 1.99). **d** Change in boreal owl clutch size from previous year (Table S8, n = 106 nests, 55 boxes, 27 years, ΔAIC = 1.16). ΔAIC denotes the difference in AIC from the highest-ranked model among those that included the variable representing the proxy for microtine abundance. All models included in this analysis are described in Tables S5-S8. Generalized linear mixed-effect models with log link function, binomial distribution, and Laplace approximation to the likelihood. Explanatory variables are standardized. For the ordered factor "Change owl breeding", "L" denotes the linear term, evaluating a straight-line relationship, while "Q" denotes the quadratic term, capturing non-linear patterns.
